# Computer Based Correlation of the Texture of P63 Expressed Nuclei with Histological Tumour Grade, in Laryngeal Carcinomas

**DOI:** 10.1155/2014/963076

**Published:** 2014-12-14

**Authors:** Konstantinos Ninos, Spiros Kostopoulos, Ioannis Kalatzis, Panagiota Ravazoula, George Sakelaropoulos, George Panayiotakis, George Economou, Dionisis Cavouras

**Affiliations:** ^1^Department of Physics, School of Natural Sciences, University of Patras, Rio, 26504 Patras, Greece; ^2^Medical Image and Signal Processing Laboratory, Department of Biomedical Engineering, Technological Educational Institute of Athens, Ag. Spyridonos, Egaleo, 12210 Athens, Greece; ^3^Department of Pathology, University Hospital of Patras, 26504 Rio, Greece; ^4^Department of Medical Physics, School of Health Sciences, Faculty of Medicine, University of Patras, Rio, 26504 Patras, Greece

## Abstract

*Background*. P63 immunostaining has been considered as potential prognostic factor in laryngeal cancer. Considering that P63 is mainly nuclear stain, a possible correlation between the texture of P63-stained nuclei and the tumor's grade could be of value to diagnosis, since this may be related to biologic information imprinted as texture on P63 expressed nuclei. *Objective*. To investigate the association between P63 stained nuclei and histologic grade in laryngeal tumor lesions. *Methods*. Biopsy specimens from laryngeal tumour lesions of 55 patients diagnosed with laryngeal squamous cell carcinomas were immunohistochemically (IHC) stained for P63 expression. Four images were digitized from each patient's IHC specimens. P63 positively expressed nuclei were identified, the percentage of P63 expressed nuclei was computed, and 118 textural, morphological, shape, and architectural features were calculated from each one of the 55 laryngeal lesions. Data were split into the low grade (21 grade I lesions) and high grade (34 grade II and grade III lesions) classes for statistical analysis. *Results*. With advancing grade, P63 expression decreased, P63 stained nuclei appeared of lower image intensity, more inhomogeneous, of higher local contrast, contained smaller randomly distributed dissimilar structures and had irregular shape. *Conclusion*. P63 expressed nuclei contain important information related to histologic grade.

## 1. Introduction

Laryngeal cancer amounts to about 3% of new diagnosed human cancers [1] and it has bad prognosis. Laryngeal cancer has been related to a number of risk factors such as smoking, alcohol drinking, heredity, and environmental substances [2–6]. Considering that in Europe 55% of the patients with laryngeal cancer may survive more than five years after first diagnosis [7], early and accurate diagnosis is important for adopting the right treatment and, consequently, for improving patient survival. Regarding accurate diagnosis, evaluation of the tumor's severity or grade is routinely performed by examining, on a conventional light microscope, tumor biopsy specimens stained with hematoxylin and eosin (H&E). However, grading of laryngeal lesions is influenced by the examining physician's experience and by low inter- and intraobserver reproducibility rates [8–10] amongst experienced pathologists. Regarding early diagnosis, many researchers have focused on identifying new prognostic factors, one such factor being the production of P63 protein by the TP63 gene, and the association of P63 overexpression with epithelial neoplasms of the lanynx [11–14]. It has been found [11] that in 96% of patients with laryngeal squamous cell carcinomas, the immunoexpression of P63 was present in over 30% of the cells, while in another study [14] the P63 cell-immunostaining cut-off was set at 50% and it was expressed in 95% of the patients with esophageal squamous cell carcinoma.

While P63 has been mostly used as a potential prognostic factor, it is also considered as nuclear stain. On the other hand, H&E, which is primarily used for histological grade assessment of laryngeal cancer, is not a predominantly nuclear stain. Thus, the question arises if there might exist a correlation between the nucleus texture of P63-expressed nuclei and the tumor's grade, as determined by H&E staining. Such a finding would be beneficial to improving diagnosis of laryngeal cancer, since this may be related to biologic information imprinted as texture on P63 expressed nuclei. However, for such an association to be investigated, a computer-based analysis of the texture of P63 stained nuclei would be required.

Computer-assisted image analysis methods have been previously utilized to automatically analyze digitized laryngeal histopathology H&E stained images, with speed and accuracy, in the effort to assess patient survival rates or to identify image parameters that differentiate between different types of lesions. Delides et al. [15] evaluated the fractal dimension, which reflects textural heterogeneity, on H&E stained histology images of laryngeal tumor tissues and found that patients with lower values of fractal dimension had higher survival rates. Dobroś et al. [16] used image analysis methods on H&E stained histopathologic sections of patients with laryngeal carcinomas for evaluating cell parameters, such as cell concentration, nuclear area, perimeter, density, roundness, and associated parameter thresholds with patient survival. In another study regarding laryngeal cancer [17], Galectin-3 labeled digitized microscopy images of patients with in situ laryngeal carcinoma, laryngeal invasive squamous cell carcinoma, and cervical lymph nodes were analyzed by a commercial software. Image analysis consisted of cell segmentation and automatic cytoplasm counting for automatically evaluating Galectin-3 expression. High Galectin-3 expression was related to the invasiveness and aggressiveness of laryngeal carcinomas. In a study by Dreyer et al. [18], computer analysis of images was employed to automatically calculate the fractal-area feature as a basis for discriminating between benign, dysplastic, and malignant squamous epithelium of the larynx. Teresa et al. [19] performed a computer-based analysis of AgNOR and Ki-67 stained oral biopsy specimens, by automatically counting positively Ki-67 stained cells and by evaluating a number of AgNOR morphometry parameters, in order to assess the proliferative status of oral epithelial cells in oral cancer. A good account on algorithms used in computer-assisted numbering of cancer cell nuclei in histological sections may be found in [20, 21].

Furthermore, computer-based decision support systems have been designed to function as second opinion tools in characterizing laryngeal cancer lesions [22, 23], employing powerful pattern recognition techniques on microlaryngoscopy color images. In a recent study by our group [24], a high precision decision support system was designed, employing immunohistochemically stained for p63 expression histopathology images, for discriminating between low from high grade laryngeal lesions.

In the present study, digitized images of histologic sections from laryngeal cancer lesions, immunohistochemically stained for P63 expression, were used to analyze, by computer processing, the amount of P63 staining and the texture of P63 expressed nuclei and relate them to the histologic grade of the lesion. The grade of laryngeal lesions had been previously determined by an experienced pathologist by means of H&E stained digitized images of specimens from the same laryngeal lesions. A number of textural and morphologic features were computed from the P63 expressed nuclei. Computer analysis revealed textural properties that differed significantly between the grades of laryngeal cancer. This constitutes new important information related to histologic grade. Additionally, a nonlinear prediction equation was designed, employing as equation parameters the percentage of P63 staining and a few textural and morphologic features. That equation would estimate the probability of a P63 stained laryngeal tumor biopsy specimen for being of low or high grade. To our knowledge, similar study on immunohistochemically stained for P63 expression laryngeal cancer biopsy specimens that relates texture and morphology properties of P63 stained nuclei to histological grade has not been previously published.

## 2. Material and Methods

### 2.1. Clinical Material

Archival material from fifty five patients with diagnosed laryngeal cancer, who had undergone biopsy between the years 2008 and 2012, was collected by an experienced histopathologist (P.R.) in the Department of Pathology, University Hospital of Patras, Rio, Greece. Patients had a mean age of 63, ranging between 44 and 89 years old, and most were smokers (47/55). Laryngeal lesion sites (see Table 1) were 35 glotic and 11 supraglottic, 3 were spread to more than one laryngeal subsite, and for 6 patients information related to lesion site was not filed. Clinical and pathological staging were determined following the American Joint Committee on Cancer (AJCC) guidelines [25]. There were eight T2, twenty-nine T3, and thirteen T4 cases. There were forty-three N0 cases, two N1 cases, and five N2 cases. Seven cases were stage II, twenty-seven stage III, and sixteen stage IV. For five cases the TNM evaluation (*T: tumor size, N: lymph nodes involvement,* and* M: distant metastasis*) was not filed. All lesions were diagnosed as laryngeal squamous cell carcinomas.

Biopsy sections were formalin-fixed and paraffin embedded, and the specimens were hematoxylin and eosin (H&E) stained for histological tumour grading and stage assessment, and immunohistochemically (IHC) stained for P63 expression. Thus, for each case H&E and P63 stained specimen tissues were generated. Twenty-one cases were diagnosed as grade I, eighteen as grade II, and sixteen as grade III. The material was retrospectively retrieved from the archives of the Histopathology Department. Tumor grade was assessed by an experienced histopathologist (P.R.) on the H&E stained specimens, following routine visual assessment under the microscope [26]. At the time of conducting the present study, the quality of H&E slides was checked by the histopathologist and few cases were omitted from the initial material due to muddy and/or uneven stain. Furthermore, P63 expression was assessed by visual inspection on the IHC-stained specimens by the same histopathologist, who set the threshold at 50% (regarding the percentage of positively expressed nuclei present in the whole of the slide under examination) for considering a case as having positive P63 expression. Below that threshold cases were not considered and were removed from the material of the present study. P63 staining was visually assessed by the same physician who at the same time marked regions on the substrate of the slide, indicating regions of interest for subsequent computer based image analysis. During the IHC evaluation the physician did not take under consideration the case's histological grade. Finally, a total of 55 cases were considered suitable and were used for further processing.

### 2.2. Computer-Based Analysis

From each case, four nonoverlapping images were selected from IHC-stained specimens regions that the pathologist had outlined on the substrates. Images were digitized at ×400 magnification, using a Leica DM2500 light microscope equipped with a Leica DFC420C digital camera, connected to a PC, with image resolution of 1728 × 1296 × 24 bits. The accompanying imaging software regulated automatically image capture parameters, such as exposure time, image contrast, image amplification, gamma value, and white balance.

**Figure 1:**
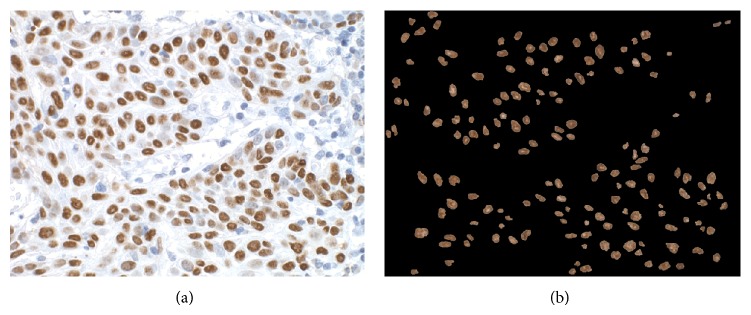
(a) Digitized frame from P63 stained specimen and (b) P63 expressed nuclei.

The first stage of the computer analysis consisted of locating the nuclei present in each image of the patient, delineating their outline, and calculating the P63 positively expressed nuclei as a fraction (%P63) of the total number of nuclei present in the patient's four images. The segmentation method that was used in the present study for identifying the nuclei has been previously developed by our group for other similar applications and a detailed account the method may be found in [21]. Briefly, the segmentation algorithm comprised two stages, first an image preprocessing stage, in which the original digitized image was transformed from a three-colour RGB image into a two-colour *L* ^*^ *a* ^*^ *b* ^*^ [21] image, for easier processing (*L* ^*^: light and dark intensities differences, *a* ^*^: redness-greenness differences, *b* ^*^: blueness-yellowness difference). In the second stage, the fuzzy C-means clustering algorithm divided the *L* ^*^ *a* ^*^ *b* ^*^ image into three pixel clusters, the expressed nuclei pixels cluster, the unexpressed nuclei pixels, and the background pixels cluster, which belonged to the surrounding the nuclei tissues. Two images were, thus, formed one from the expressed and the other from the nonexpressed pixels only, which were displayed with their original RGB value. Nuclei in the first image attained brownish colour (expressed nuclei) and in the second image bluish colour (nonexpressed nuclei). Both images were next processed by morphological and size filters to complete the shape of the nuclei and to discard formations less than 300 pixels in size; 300 was the lower threshold set for identifying a formation as nucleus and it was set experimentally. Figures 1(a) and 1(b) present the original and segmented (P63 expressed) nuclei images. The correctness of segmentation was visually evaluated by the experienced pathologist by examining the nuclei in the segmented and the original images.

The second stage of the computer analysis consisted of evaluating a large number of textural and morphological features from each one of the P63-expressed nuclei and in each one of the lesions's four images. Each lesion was, thus, represented by one feature-vector, containing one hundred and eighteen feature averages. Three classes were, then, formed each containing the feature-vectors of the corresponding to grade I, grade II, or grade III cases. Next, each feature's discriminatory power, in terms of statistical significance differences between laryngeal lesion grades, and each feature's correlation to laryngeal tumour grade advancement were estimated. This is expected to produce useful information regarding the change of nuclei texture and shape with disease severity. Textural features were generated using the gray-scale version of the P63 expressed nuclei. Four features were calculated from the nucleus-image histogram (mean value, standard deviation, skewness, and kurtosis). Twenty six (26) features were extracted from the nucleus-image's cooccurrence matrices [27] (in fact, 13 features, each represented by two feature-values, the average and range over four directions). Ten features were evaluated from the nucleus-image's run-length matrices [28] (actually, 5 features, each represented by two feature-values, the average and range over four directions). Twelve features were calculated from the frequencies of the 3-bin histograms of the nucleus-image Radon transform at 0, 45, 90, and 135 degrees [29, 30]. Twenty-four (24) features were estimated from the frequencies of the nucleus-image multi-scale histograms at 3, 5, 7, and 9 bins [31]. Six Tamura [32] textural features were estimated, regarding nucleus-image coarseness, directionality, and contrast and the three frequencies of the 3-bin histogram. Eleven edge statistics [33] features were formed by convolving the nucleus-image with the Prewitt gradient and calculating the mean, median, variance, and the frequencies of the 8-bin histogram of the processed nucleus image. Since P63 predominantly stained the nuclei, it meant that nuclei were better defined within the surrounding matter and this led us to consider additional nuclear features. Ten features were calculated from the morphology of the nuclei (area, perimeter, eccentricity, length major axis, length of minor axis, convex area, solidity, equivalent diameter, rectangularity, and compactness). Six features evaluating the shape of the nucleus outline were computed [34] (mean, standard deviation, range and entropy of the radial distance from the nucleus centroid, circularity ratio or circle variance, which is defined as the ratio of the mean over the standard deviation of the radial distance, and outline roughness index). The spread or physical topology of the nuclei in the image was evaluated employing architectural features. Those features were derived from the minimum spanning tree (MST), which is formed by joining all nuclei in the image with the minimum length. Seven such features were evaluated by measures of the MST (mean, range, standard deviation, maximum, minimum, sum, and total length). Additionally, the fractal dimension, which evaluates heterogeneity of tumour tissue and has been shown to be a prognostic factor in laryngeal carcinoma [15], was evaluated. Finally, the percentage of P63 expressed nuclei in the patient's digitized images was calculated, since it has been previously associated with patient prognosis [11].

Textural features from the nucleus image histogram, the cooccurrence, and run-length matrices were evaluated as described in a previous study by our research group [21]. Features from the nucleus morphology, the MST, and the fractal dimension were evaluated from functions available in the Matlab software. The rest of the features (Radon transform, multiscale histograms, Tamura descriptors, edge statistics, and shape features) were calculated in accordance with previous studies [29–34]. Each case was, thus, represented by a 118-dimensional feature vector, in which each feature was the average calculated from all P63-expressed nuclei detected in the case's four digitized images.

The third stage of the computer analysis consisted of determining textural features that sustained statistically significant differences between the grades of laryngeal cancer, employing Wilcoxon statistical test, when considering two grade comparisons, and the KruskalWallis test for 3 grade comparisons [35]. The correlation between each feature and the tumour's grade was evaluated employing the Point Biserial Correlation (for feature values against distinct grades) and the correlation amongst features was estimated by employing Spearman's correlation [35].

Finally, the fourth stage of the computer analysis consisted of using the logistic regression analysis [35] to construct an equation, using nuclei properties (features), for assessing a laryngeal lesion's probability of being of either low or high histological grade of laryngeal cancer. The nonparametric Receiver Operating Characteristic (ROC) curve was used for estimating the discriminating power of the nonlinear logistic regression model, by means of the Area Under the ROC curve (AUC). First, data were normalized to zero mean and unit standard deviation by means of ${\overset{-}{f}}_{i}=\left(f_{i}-m\right)/\sigma$, where ${\overset{-}{f}}_{i}$ is the normalized version of feature *f*_*i*_ and *μ* and *σ* are the mean and standard deviation of feature *f*_*i*_, both calculated over all patterns of both classes. This measure was taken in order to avoid feature-value bias. Second, a feature-vector of high discriminatory power was selected, amongst the 118 features, in order to construct the nonlinear logistic regression equation. This was accomplished by means of the Sequential Forward Floating Selection (SFFS) feature selection technique [36], employing a class separability measure, and the leave-one-out (LOO) cross-validation method [36] for a less biased assessment of the equation's discriminatory power. The class-separability function was calculated by means of *J* = trace  {*S*_*w*_^−1^ *S*_*m*_}, where *S*_*w*_ is the within-class scatter-matrix, *S*_*m*_ is the feature vector covariance matrix, and trace refers to the sum of the main diagonal matrix terms. Most of the above statistical test procedures are available as functions in the Matlab software and/or in [37].

## 3. Results

In the image segmentation stage, about 87%, on average, of the segmented objects were recognized by the physician as nuclei. This is not far from the findings of previous studies [21, 38–41]. The physician had to inspect segmented images against the original RGB images of each patient, with the task of identifying and excluding from the images segmented objects that were not nuclei. This was accomplished by means of the custom developed software. Those segmented images, containing only the verified positively and negatively expressed nuclei, were used for further processing.

Examining the values of nuclei textural features and how these values may change with advancing grade, it was found that at the 1% (*P* = 0.01) statistical level there were two features that displayed statistical significant difference (SSD) amongst the three grades; the Long Run Emphasis (LRE) and the Run Percentage (RP) textural features from the run length matrix.

**Figure 2:**
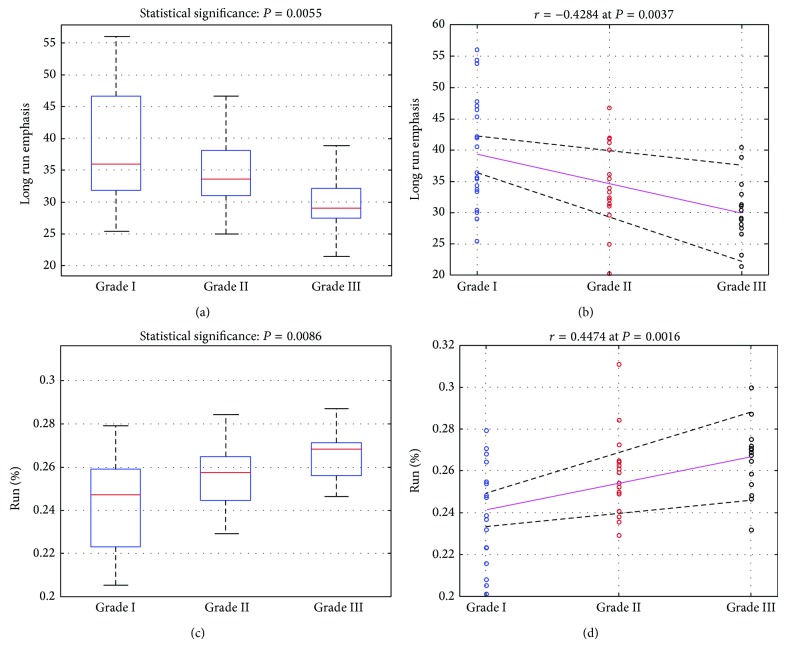
Box plots and correlation plots of the Long Run Emphasis ((a) and (b)) and Run Percentage ((c) and (d)) features, respectively, sustaining statistically significant differences (*P* < 0.01) between the three laryngeal grades.

Regarding the Long Run Emphasis textural feature, Figure 2(a) shows the boxplots of the three grade-classes, depicting, at each grade, the spread, and median of the feature values. LRE revealed SSD amongst the three grade classes of *P* = 0.006 and negative correlation of *r* = −0.42 at a confidence level (probability for the null hypothesis to hold) of *P* < 0.005 (*P* = 0.004). Examining the between the grade-classes SSDs of the LRE feature, it was found that only grade I and grade III classes sustained SSD (*P* = 0.0008), while grade II and grade III class comparisons showed no SSD at the 1% statistical level.  Figure 2(b) shows the point biserial correlation of the RLE feature with advancing grade and the 95% confidence levels.

SSDs amongst the three grade classes at the 1% statistical level were also revealed by the Run Percentage textural feature.  Figure 2(c) shows the boxplots of the three classes for the RP feature, sustaining SSD amongst grade classes of *P* < 0.01 (*P* = 0.009) and positive correlation of *r* = 0.45 at statistical confidence level of *P* < 0.005 (*P* = 0.002). Examining the between classes SSDs of RP, it was found that grade I class sustained SSD with grade III class (*P* = 0.01) and that there was no SSD between grade II and grade III class-comparison at the 1% statistical level.  Figure 2(d) shows the point biserial correlation of the RP feature with advancing grade and the 95% confidence levels.

Since both LRE and RP features showed no SSDs between grade II and grade III classes and since non-SSDs were also verified in the overwhelming majority of the examined features, it was decided to combine grade II and grade III classes into one class, the High Grade class. Thus, from here on, a two-class problem is considered, consisting of the low grade (LG) class, containing the grade I laryngeal tumour cases, and the high grade (HG) class, comprising the grade II and grade III laryngeal tumour cases.

**Figure 3:**
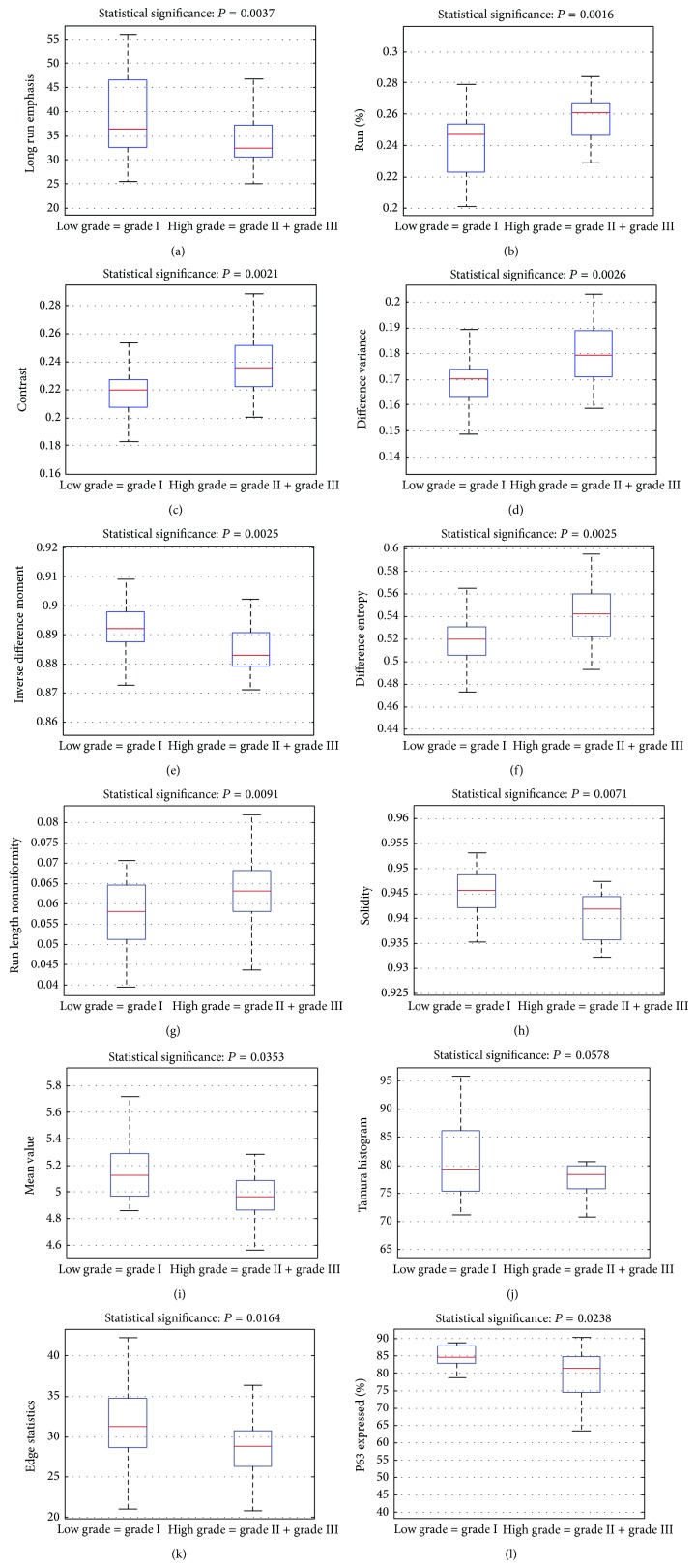
Box plots of features sustaining statistically significant differences between low and high grade classes. (a) Run length emphasis, (b) run percentage, (c) contrast, (d) inverse difference moment, (e) difference variance, (f) difference entropy, (g) run length nonuniformity, (h) solidity, (i) mean value, (j) Tamura histogram feature, (k) edge statistics feature, and (l) percentage of positively expressed nuclei.

In the LG against HG class comparisons, six more textural features showed SSDs at the 1% level as well as correlations at good confidence levels; contrast, inverse difference moment, difference variance, difference entropy, run length nonuniformity, and solidity. The first four features were calculated from the cooccurrence matrix, the fifth from the run-length matrix and the sixth from the morphology of the nuclei. As shown in Figure 3 and Table 2, all eight features had SSDs between the LG and HG classes and correlations with advancing grade either positive or negative. Additionally, by relaxing the statistical threshold to *P* < 0.05, which is well accepted statistical level in medical studies, four more features were found to sustain SSDs between LG and HG laryngeal lesions, the mean value, the percentage of P63 expressed nuclei, the Tamura histogram feature (third component of the 3-bin coarseness histogram), and the edge statistics feature (the 8th component of the 8-bin histogram).

Table 2 shows, for each one of the 12 features, the mean values and standard deviations of each feature as well as the SSDs between the LG and HG classes and the point biserial correlations at statistically significance level (at least *P* < 0.05 or smaller). In more detail and as shown in the boxplots in Figure 3 and the values of Table 2, LRE and RP features both sustained SSDs at *P* < 0.005 and correlations of −0.43 and 0.44, respectively. Similarly, the contrast (CONT) feature sustained SSD between LG and HG classes at *P* < 0.01 and *r* = 0.44, the inverse difference moment (IDF) feature SSD at *P* < 0.01 and *r* = −0.43, the difference variance (DVAR) feature SSD at *P* < 0.01 and *r* = 0.43, the difference entropy (DENTR) feature SSD at *P* < 0.01 and *r* = 0.43, the run length nonuniformity (RLNU) feature SSD at *P* < 0.01 and *r* = 0.35, the solidity feature SSD at *P* < 0.01 and *r* = −0.35, the mean value (MV) feature SSD at *P* < 0.05 and *r* = −0.32, Tamura histogram feature (TamuraH) SSD at *P* < 0.05 and *r* = −0.3 and the edge statistics feature (EdgeSt) SSD at *P* < 0.05 and *r* = −0.35.

Regarding the percentage of P63 expressed nuclei, the particular parameter was calculated for each patient automatically by computer processing of the 4 patient images, and it referred to the percentage of P63 expressed nuclei over the total number of nuclei detected within the areas of interest, which were indicated by the expert physician. As seen in Figure 3(j) and Table 2, it was found that the percentage of P63 expressed nuclei in the two classes differed significantly at the 5% level (*P* = 0.02) and displayed a negative correlation of decreasing P63 expression with advancing grade (*r* = −0.3). This correlation was significantly different from zero at *P* < 0.05 level of confidence. That is, the percent of P63 expressed nuclei decreases with advancing grade.

**Figure 4:**
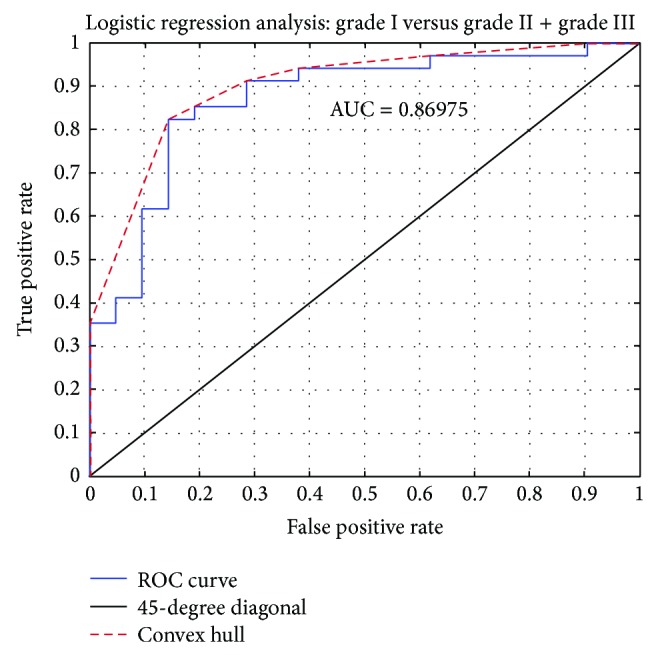
ROC curve: using best features combination in the logistic regression equation and the LOO cross-validation method, highest prediction accuracy was achieved (AUC = 0.87, features involved: %P63 expressed nuclei, Angular Second Moment^*r*^, Correlation^*a*^, Inverse Difference Moment^*r*^, Sum Entropy^*r*^, Solidity, and Radial Distance range). *a*: average and *r*: range of the feature over four directions (0°, 45°, 90°, and 135°).

Although the features described above showed SSDs between LG and HG classes, that is, they possessed reasonably high discriminatory power between the two classes, however, when examined individually it was realized that they could not be used to assess the probability of a case as belonging to either the low or the high grade classes. This becomes clear when one examines the mean values and standard deviations in Table 2 and the spread of values in the boxplots of Figure 3; features of good discriminating power and with valid, positive, or negative correlation, had their feature values distributed with an evident overlap between the two classes. This means that one would hesitate to propose an index, based on the values of an individual feature, which would assign one case to either low or high grade class, with reliable prediction accuracy. For this to be feasible, a number of features would have to be combined, linearly or non-linearly, into one equation, which hopefully would draw apart the multifeature distributions of the two classes and, thus, form a multidimensional index, to be used as a grade prediction model. Those features, however, would have to be as much uncorrelated as possible, if classes were to be distinctively separated [36]. Following feature normalization to zero mean and unit standard deviation, it was found that, in their majority, those features bared high correlations amongst themselves. Thus, when they were examined in various combinations, as parameters in the nonlinear logistic regression equation, the so formed grade prediction model was of low prediction credibility. Searching for other feature combinations, by looking into the set of 118 generated features in the present study, of up to seven features (a rule of thumb is that features in a combination should not exceed 1/3 of the cases in the smallest class [36]), it was found that the following features combination (or vector) provided highest prediction accuracy: percentage of P63 expressed nuclei, Angular Second Moment (range), correlation (average), Inverse Difference Moment (range), Sum Entropy (range), Solidity, and Radial Distance (range). That best-feature vector was determined by means of the SFFS feature selection method. Its prediction accuracy was evaluated by means of the leave-one-out cross-validation method and it was found 85.5% (see Table 3); 16 out of 21 LG cases and 31 out of 34 HG cases were predicted correctly. Table 3 also shows the index value (0.654) of the Cohen-Kappa test statistic, which is rated as a “good” indicator that the result was not achieved by chance. Employing the ROC curve, as an estimate of class separability, the particular feature-combination, when used in the nonlinear logistic regression equation, resulted in an (area under the curve) AUC = 0.87, by employing the LOO cross-validation method. This AUC is rated as “very good” and it assesses the ability of the particular equation in predicting the grade of a laryngeal lesion (see Figure 4). The nonlinear logistic regression equation, using the values of those 7 features, was formed as follows:

(1) $$\begin{array}{c} P\left(f_{i}\right)=\frac{1}{\left(1+e^{-Fw_{i}}\right)}, \end{array}$$

where *P* is the probability assigned to a particular pattern; if *P* > 0.5 then the particular pattern is assigned to the HG class, otherwise to the LG class where

(2) $$\begin{array}{c} Fw_{i}=\overset{7}{\underset{i=0}{\sum }}w_{i}*f_{i} \end{array}$$

and  *w*_*i*_ = {1.63, −3.96, 30.06, 10.37, −7.32, −22.25, −2.52, −1.38} for *i* = 0,1,…, 7 are weights of the constructed discriminant function and *f*_*i*_ = {1, “%P63* expressed nuclei*”, “*Angular Second Moment* ^*r*^”, “*Correlation* ^*a*^”, “*Inverse Difference Moment* ^*r*^”, “*Sum Entropy* ^*r*^”, “*Solidity*”, and “*Radial Distance range*”} represent the actual numerical values of the particular normalized features vector in the equation, and where *a* and *r* stand for average and range of the feature over four directions (0°, 45°, 90°, and 135°).

## 4. Discussion

Textural, morphology or shape properties of cell-nuclei have been previously employed in the analysis of microscopy images. These properties, in the form of numerical features, have been used for improving diagnosis, prognosis, and eventually management of various types of tumours, such as breast [42, 43], thyroid [44], brain [45, 46], endometrium [47], larynx [15, 17, 18], and lung [48]. The aim of the present study was to analyse by computer processing microscopy images of biopsy material from laryngeal cancer lesions, which had been stained for P63 expression. P63 targets mainly the nuclei, in contrast to H&E that stains the surrounding the nucleus matter, too. It is, thus, of importance to identify the nuclei in the P63 stained image, to analyse the nucleus properties, regarding texture, shape, and morphology and to investigate if some of those properties may be related to tumour progression and to changes in the nucleus structure with advancing grade. Such an approach, employing P63 staining methods to analyse the laryngeal tumour nucleus, to our knowledge, has not been previously published.

Data of the present study were histologically graded into three distinct classes (grades I, II, and III) by an experienced histopathologist, employing the H&E staining routine examination and the WHO rules for grading. Data were also stained for P63 expression. Regarding textural features, the most important features found were the Long Runs Emphasis and the Run Percentage. Both features displayed statistical significant differences amongst the three classes, when examined by the Kruskal-wallis statistical test (Figure 2). Those differences were due to the statistical strong differences sustained, for both features, between classes of grade I and grade III, while there were no statistical differences found between the other classes.

The Long Run Emphasis textural feature reflects the presence of large formations in texture. It was found that the LRE-values in the low grade class were higher than in the higher grade classes (Figure 2(a)) and this was reflected in the negative correlation shown in Figure 2(b). This probably indicates a break down in the structure of the texture of the high grade P63-stained nuclei, which in turn indicates the destruction of P63 receptors at advanced grade, resulting in the dismantling of textural content and loss of continuum in image structures. This is in line with the fact that high grade cells are poorly differentiated and lack normal tissue cell structure [25]. Similar findings were also found, with regards to the LRE feature, in the two class comparisons (i.e., LG versus HG), as it may be seen in Table 2 and Figure 3(a).

The Run Percentage is a textural feature that assesses the number of different structures existing in the image and it attains low values when this number is small, indicating that the image is mostly of linear structure. As shown in Figure 2(c), the RP feature sustained statistically significant difference amongst the three grade-classes at *P* < 0.001, having a positive correlation of *r* = 0.547 at a confidence level of *P* = 0.0005. This finding indicates that the texture of high grade P63 stained nuclei tends to be more inhomogeneous and it appears to contain more different structures. Most probably, the loss of continuum in stained nucleus texture, due to the destruction of P63 receptors at advance histological grades, results in the formation of a multitude of microstructures of various sizes and, thus, in a nucleus texture of increased inhomogeneous appearance and loss of image linearity. Similar findings were also found, regarding the RP feature, in the two class comparisons (i.e., LG versus HG), as it may be seen in Table 2 and Figure 3(b).

By splitting data in two classes, the LG class containing grade I cases and the HG class consisting of the grade II and III cases, six more textural features were found to sustain statistically significant differences at the 1% level between the two classes: contrast, inverse difference moment, difference variance, difference entropy, run length nonuniformity, and solidity and four more at the *P* < 0.05 level, the mean value, the percentage of P63 expressed nuclei, the Tamura histogram feature, and the edge statistics feature.

The Contrast feature is a measure of image contrast. It evaluates the amount of local variations present in the image texture, and it attains high values for large amounts of local variations. As it may be observed from Figure 3(c), high grade nuclei display higher local variations that probably lead to patchy staining of P63 nuclei. This may be explained by considering the destruction of P63 receptors in high grade nuclei, which contributes to randomness in the location of the remaining P63 receptors within the nucleus texture, thus resulting in increased variations in the staining of the nucleus texture across the nucleus image.

The Inverse Difference Moment feature is a measure of image homogeneity, and it attains larger values for smaller gray-tone variations. It was found that high grade P63 expressed nuclei had smaller IDF values (see Figure 3(d)), which translates into higher gray-tone variations across the nucleus image. This finding indicates that P63 staining in high grade nuclei was more inhomogeneous and that P63 stain homogeneity decreased with advancing laryngeal grade. This may again be attributed to the dismantling of the high grade nuclei and to the subsequent destruction of P63 receptors.

The Difference Variance feature is a measure of variation in image contrast. It attains low values for equally distributed contrast transitions. As shown in Figure 3(e), DVAR attained larger values in high grade P63 stained nuclei, indicating that high grade nuclei images contained many unequally distributed local variations in the P63 stained nuclei.

The Difference Entropy is a measure of randomness or lack of structure or order in the image contrast. It attains high values in randomly distributed image gray-tone differences. As it can been seen in Figure 3(f), DENTR increased with advancing grade. This indicates that the P63 stained texture of high grade nuclei has more unstructured distribution of image contrast.

The Run Length Nonuniformity feature measures nonuniformity of the run lengths and it attains low values if structures (run-lengths) are equally distributed throughout the lengths. As can be observed from Figure 3(g), RLNU increased with advancing grade. This finding indicates that the size of formations or structures within the P63 stained nuclei texture differed and was unevenly distributed in the HG class.

The Solidity feature is a measure of nucleus shape convexity, it is calculated by the ratio of the nucleus area over the area of the smallest convex hull polygon that can contain the nucleus, it indicates nucleus outline uniformity and it decreases with increasing nuclei boundary irregularity. As can be observed from Figure 3(h), solidity decreased with increasing grade and this suggests that P63 expressed nuclei tend to attain irregular shapes at high grades.

The mean value feature is a measure of the nucleus texture intensity and it is related to the existence of P63 receptors in the nucleus. It can be seen from Figure 3(i), the nuclei in the HG class were of lower intensity. This again is most probably attributed to the destruction of P63 receptors as a result of the nucleus break-down at high grades.

The Tamura histogram feature, which refers to the third constituent of the 3-bin coarseness histogram, reflects the frequency of large components (primitives) in the nucleus image. HG nuclei are expected to have fewer large components, because of the destruction of P63 receptors, leading to smaller values in the Tamura feature (see Figure 3(j)) and to coarser nucleus texture due to the existence of relatively smaller primitives.

The edge statistics feature, which is the eighth component of the eight bin magnitude histogram, echoes the frequency of high intensity pixels in the nucleus texture. As shown in Figure 3(k), it decreased at higher grades, which is indicative of lowering intensity in HG nuclei that may be attributed to the damage of P63 receptors at high grades.

Regarding the positive immunoexpression of P63, it was found that the percentage of P63 (%P63) stained nuclei decreased with advancing histological grade (see Figure 3(l)). It was found that between low grade and high grade cases there was a negative correlation at a confidence level of *P* < 0.05 (see Table 2), meaning that P63 expression was found lower in the high grade nuclei. It was also found that P63 staining was statistically different between the low grade and high grade cases at *P* < 0.05. This may be due to alterations in the structure of cell nuclei in high grade tumours that may have a destructive effect on the P63 receptors, which are responsible for the P63 staining of the cell nucleus. Such finding, that relates advancing laryngeal histology grading to diminishing P63 staining, has not been previously reported. However, since high grade laryngeal tumours are usually associated with bad prognosis, there have been previous published studies that relate P63 expression to prognosis. In previous study [11], it has been found that decreased immunoexpression of P63 in grade II laryngeal squamous cell carcinomas was related to the risk of recurrence and death by cancer and that it has bad prognosis. On the contrary, in another study [12] the opposite has been claimed while other studies [13, 49] have not found any association among P63 expression and prognosis.

As it is made obvious, several of the above textural features evaluate similar aspects of textural properties, such as image intensity, contrast, homogeneity, and content. However, for complicity, those features had to be described as to the textural properties they express, as part of the analysis of P63 staining of the laryngeal cancer nuclei across histological grades.

Regarding the need for predicting the grade of a laryngeal lesion, one would be tempted to use combinations of those features of high discrimination power in the formulation of the logistic regression model. However, such an attempt would probably fail to produce high discriminative models, since those features are highly correlated with each other [36]. In fact, the ROC curve of such an attempt resulted in AUC = 0.77, found after experimentation with all possible combinations within the 12 highly discriminative features, employing the LOO method. Such an outcome, however, may be rated as of “low” discriminatory ability. On the other hand, when a search by the SFFS method for up to 7 features combinations within the available 118-features was performed, there was one feature-combination that produced a ROC curve (see Figure 4), which was rated as of “very good” discriminating ability (AUC = 0.87), using the LOO evaluation method. This feature combination comprised the percentage of P63 expressed nuclei, the Angular Second Moment (range), the Correlation (average), the Inverse Difference Moment (range), the Sum Entropy (range), the Solidity, and Radial Distance range. The Angular Second Moment (range) evaluates the anisotropy in nucleus-image homogeneity along four directions (0, 45, 90, and 135 degrees), the Inverse Difference Moment (range) estimates the anisotropy in image gray-tone differences, the Correlation (average) measures the gray-tone linear-dependencies in the nucleus-image, the Sum Entropy (range) estimates the anisotropy in randomness or lack of structure or order in the nucleus-image, the Solidity expresses the convexity of the nucleus shape, and the Radial Distance range evaluates the irregularity of the nucleus outline. Of those best features, only the percentage of P63 expression and the Solidity showed statistical significant differences between the two classes. Those two features displayed negative correlation with increasing grade, indicating that high grade nuclei appear less stained and of irregular shape.

Summarizing, it was found that with advancing grade, P63 staining of laryngeal lesion nuclei decreases with subsequent lowering of nucleus image intensity, the texture of the P63 stained nuclei becomes more inhomogeneous, of higher local contrast, it contains smaller dissimilar structures randomly distributed and nuclei are of more irregular shape. Prediction of the histologic grade by the logistic regression equation may be accomplished, based on P63 staining, textural, morphology, and shape features of the nuclei.

## Figures and Tables

**Table 1:** Site, stage, and grade distribution of laryngeal tumor lesions.

		High differentiation (grade I)	Moderate differentiation (grade II)	Low differentiation (grade III)	Total
		21	18	16	55

Lesion site	Glotic	17	11	7	35
	Supraglotic	3	3	5	11
	Spread to subsites	0	2	1	3
	N/A	1	2	3	6
Stage	T2	3	3	2	8
	T3	13	9	7	29
	T4	4	4	5	13
	N0	18	12	13	5
	N1	1	0	1	43
	N2	1	4	0	2
	*N/A *	*1 *	*2 *	*2 *	*5 *
	II	3	3	1	7
	III	12	7	8	27
	IV	5	6	5	16

**Table 2:** Means, standard deviations, statistical significance, and correlations of features with statistically significant differences between High Grade and Low Grade laryngeal tumor lesions.

	LG-class		HG-class		LG versus HG	LG versus HG
	mv	std	mv	std	Statistical significance	Correlation *r* at *P* < 0.05
% P63	84.840	3.251	79.833	9.565	0.02	−0.300
MV^a^	5.170	0.260	5.010	0.211	0.03	−0.322
CONT^a^	0.216	0.024	0.238	0.02	0.002	0.436
IDF^a^	0.893	0.012	0.883	0.009	0.003	−0.434
DVAR^a^	0.168	0.014	0.179	0.010	0.003	0.434
DENTR^a^	0.515	0.033	0.542	0.023	0.003	0.434
LRE^a^	39.475	9.026	32.351	6.122	0.004	−0.428
RLNU^a^	0.057	0.008	0.064	0.009	0.009	0.354
RP^a^	0.240	0.022	0.261	0.019	0.002	0.447
Solidity	0.945	0.008	0.940	0.005	0.007	−0.332
TamuraH	70.751	7.601	65.974	7.021	0.040	−0.307
EdgeSt	32.205	5.939	28.356	4.245	0.016	−0.356

% P63: percentage of P63 expressed nuclei, MV: mean value, CONT: Contrast, IDF: Inverse Difference Moment, DVAR: Difference Variance, DENTR: Difference Entropy, LRE: Long Runs Emphasis, RLNU: Run Length Nonuniformity, RP: Run Percentage, TamuraH: Tamura histogram feature, EdgeSt: edge statistics feature, mv: mean value, and std: standard deviation, a: average of the feature over four directions (0°, 45°, 90°, and 135°).

**Table 3:** Contingency table of predicting the grade of laryngeal squamous cell carcinomas by the nonlinear logistic regression equation.

LG^+^	LG^−^	HG^+^	HG^−^	% overall accuracy	Cohen-Kappa	AUC
16	5	31	3	85.5	0.654	0.87

LG^+^ and LG^−^ refer to low grade (grade I) of correctly and incorrectly predicted carcinomas and HG^+^ and HG^−^ refer to correctly and incorrectly predicted high grade (grade II + grade III) carcinomas, respectively. AUC: area under the curve; overall prediction accuracy was estimated by the LOO cross validation method.
